# Evaluation of creatine kinase and liver enzymes in identification of severe dengue

**DOI:** 10.1186/s12879-017-2601-8

**Published:** 2017-07-21

**Authors:** Saiful Safuan Md Sani, Winn Hui Han, Mohamad Adam Bujang, Hui Jen Ding, Kiah Loon Ng, Mohd Azizuddin Amir Shariffuddin

**Affiliations:** 1 Department of Medicine, Hospital Kuala Lumpur, Jalan Pahang, 50586 Kuala Lumpur, Malaysia; 2 Biostatistic Unit, National Clinical Research Centre, 1st Floor, MMA Building, 124 Jalan Pahang, 53000 Kuala Lumpur, Malaysia; 3 Clinical Research Centre, Hospital Kuala Lumpur, Jalan Pahang, 50586 Kuala Lumpur, Malaysia

**Keywords:** Severe dengue, Creatine kinase, AST, ALT, Identification, Sensitivity, Specificity

## Abstract

**Background:**

Existing biomarkers such as AST, ALT and hematocrit have been associated with severe dengue but evidence are mixed. Recently, interests in creatine kinase as a dengue biomarker have risen. These biomarkers represent several underlying pathophysiological processes in dengue. Hence, we aimed to assess AST, ALT, CK and hematocrit in identification of severe dengue and to assess the correlational relationship amongst common biomarkers of dengue.

**Methods:**

This was a retrospective cohort study of confirmed dengue patients who were warded in Kuala Lumpur Hospital between December 2014 and January 2015. CK, AST, ALT, hematocrit, platelet count, WBC and serum albumin were taken upon ward admission and repeated at timed intervals. Composite indices based on admission AST and ALT were analyzed. Correlation coefficients and coefficients of determination were computed.

**Results:**

Among the 365 cases reviewed, twenty-two (6%) patients had severe dengue. AST and ALT were found to be good at identification of severe dengue. The AST^2^/ALT composite index was the most accurate (AUC 0.83; 95% CI 0.73 - 0.93). Optimal cutoff was 402 with a sensitivity of 59.1% (95% CI: 36.4 - 79.3%) and specificity of 92.4% (95% CI: 89.1 - 95.0%). Modified cutoff of 653 had a sensitivity of 40.9% (95% CI: 20.7 - 63.7%) and specificity of 97.4% (95% CI: 95.1 - 98.8%). Our analyses also suggested that several underlying biological processes represented by biomarkers tested were unrelated despite occurring in the same disease entity. Also, markers of plasma leakage were discordant and AST was likely hepatic in origin.

**Conclusions:**

The composite index AST^2^/ALT may be used as a marker for identification of severe dengue based on admission AST and ALT, with two choices of cutoff values, 402 and 653. AST is most likely of liver origin and CK does not provide additional value.

## What is already known about this topic?

It is already known that elevations of both AST and ALT levels are associated with bleeding and dengue hemorrhagic fever. Literature on creatine kinase in dengue is inadequate. Hematocrit and serum albumin are considered as markers of plasma leakage.

## What does this article add?

This study provides evidence that AST and ALT taken upon admission, through composite index AST^2^/ALT, are good parameters in identifying severe dengue regardless of types of severe dengue. AST is most likely of liver origin and CK does not provide additional value as a severity marker. Additionally, two markers of plasma leakage (hematocrit and serum albumin), do not correlate with each other.

## Background

Dengue infection is endemic to the tropical and subtropical regions of the world and is predicted to worsen and spread to wider latitudes [1]. In 2014, for the first time in history, the number of confirmed dengue cases in Malaysia breached the 50,000-case mark [2].

Like any other disease, dengue has biomarkers which are used to identify dengue infection, act as surrogates for underlying pathological process or identifying severity of infection. Current established biomarkers in clinical practice are hematocrit (Hct), platelet count, white blood cell (WBC), serum albumin and alanine aminotransferase (ALT). Recent studies and the ‘breakbone’ feature of dengue have sparked an interest in creatine kinase (CK). It has been shown in a small cohort study involving adult dengue patients (*n* = 39) that CK can be elevated, median 837 (range 194-3832 U/L) [3]. It has been further documented that any level of elevated CK taken after 48-96 h from onset of illness, was indeed associated with a more severe disease (ie. dengue haemorrhagic fever, DHF) by Cox proportional hazard regression analysis, HR 6.98, 95% CI 2.34 - 20.85, *p* < 0.001 [4].

Hepatitis, represented by elevated levels of aspartate aminotransferase (AST) and ALT, has a specific pattern. It is well-documented that AST is elevated to a higher level [5–9] and in a larger proportion [10–13] of patients as compared to ALT. It has also been shown that AST rises and reduces before ALT [8]. Although AST has been found to be elevated greater than ALT, its origin, musculoskeletal or hepatic, has not been proven [8, 11]. The association of transaminitis with disease severity has been found to be inconsistent. One group of investigators in Thailand [9] showed that AST but not ALT was associated with DHF, whilst another group showed both liver enzymes were significantly associated with severe cases (AST *p* < 0.001, ALT *p* = 0.003) [14]. Both AST and ALT have been claimed to be significantly correlated with clinical plasma leakage but the strengths of correlation were not reported [12]. A later study in Vietnam showed that both AST and ALT were associated with shock [8]. They found that AST and ALT levels during the critical phase were significantly higher in dengue patients who experienced shock compared to those without shock (*p* < 0.01). They also found that transaminitis correlated weakly with the nadir thrombocytopenia (Spearman *r* − 0.2 to −0.3; both *p* < 0.05). One study in Singapore showed no association between transaminases and fatality, (logistic regression analysis (fatal outcome), AST *p* = 0.14, ALT *p* = 0.11) [15], and another showed transaminases lacked the discriminatory function to classify dengue according to severity by the WHO 2009 classification (AST AUROC 0.62 (95% CI:0.57 – 0.67) and ALT AUROC 0.60 (95% CI: 0.54 – 0.64) [11]).

Widely available and practical biomarkers (elevated CK [4], liver transaminases [10–12, 14–17], Hct [17] and low serum albumin [4, 14, 17] and platelet count [12, 14, 16, 17]) have been shown to be associated with more severe disease. These biomarkers each represent a specific underlying pathophysiological process. Hct and serum albumin have long been believed to be representative of plasma leakage [18]. Platelet count and WBC have been shown to be good at reflecting disease course [19]. CK reflects muscle involvement and ALT the liver. Pathophysiological processes occurring in a single disease ought to correlate with each other. Furthermore, as these biomarkers have been shown to be associated with severe disease, a certain degree of correlation is expected amongst them. However, in our clinical observation, we have found them to be discordant with each other. Quantifying their correlation coefficients which measure the strength of those relationships, if any, may clarify their associations with each other.

Hence we endeavoured to evaluate the function of elevated CK, AST and ALT in the early identification of severe dengue. Secondly, we looked to clarify and quantify correlations amongst biomarkers: CK, AST, ALT, Hct, serum albumin, platelet count and WBC. This in turn will clarify whether AST is predominantly liver or muscle in origin.

## Methods

### Ethical considerations

The study was approved by the Malaysian Research Ethics Committee, Ministry of Health of Malaysia (Research ID  NMRR-14-1543-23635). Our report is based on the Standards for Reporting of Diagnostic Accuracy (STARD) 2015 guideline [20].

### Study design

This was a retrospective cohort study of adult patients with confirmed dengue infection who were admitted to Kuala Lumpur Hospital. Sample recruitment was conducted from December 2014 until January 2015. Sampling was universal. Inclusion criteria were patients ≥18 years old and presentation which satisfied the WHO 2009 criteria [21] for suspected dengue. In Malaysia, patients with dengue infection are admitted when they manifest severe dengue characteristics, warning signs, have underlying co-morbidities or have healthcare accessibility issues. Our centre adopts these based on recommendations of our own national clinical practice guidelines which in turn have absorbed many of WHO 2009 clinical guidelines recommendations and adopted fully WHO 2009 criteria. In addition, cases selected were required to have the presence of acute dengue viral infection confirmed via presence of NS1 antigen and/or high-titre level of IgG from an admission serum sample. Cases were excluded if they: 1) had underlying liver diseases, or 2) had received recent (within 5 days) intramuscular injections. Cases were subsequently classified into severe and non-severe dengue.

Case notes were reviewed and data, including baseline characteristics, clinical parameters and laboratory findings, were collected using a pre-defined data collection form. All patients received standard management adhering to the 2010 Malaysian Clinical Practice Guideline on Management of Dengue Infection in Adults [22] and the 2009 WHO clinical practice guideline for dengue [21]. CK, AST, ALT, Hct, platelet count, WBC and serum albumin were taken upon ward admission. As per the recommendations in the guidelines, full blood count was repeated at 8-hourly or 6-hourly intervals. Other blood investigations were repeated at least daily or more frequently as required.

### Case definitions for dengue

Case definitions for the diagnosis of dengue infection and classification of disease severity were based on the 2009 WHO guidelines. According to that system, a suspected dengue case is defined by the presence of fever plus any two of 1) aches and pain, 2) nausea and/or vomiting, 3) rash, 4) leucopenia, or 5) presence of any warning signs. Warning signs are defined as any one of: 1) abdominal pain or tenderness; 2) persistent vomiting (defined as vomiting a minimum of 3 times within 24 h); 3) non-physiological or supraphysiological bleeding; 4) lethargy; 5) clinical fluid accumulation that manifest as pleural effusion, ascites 6) a palpable liver 2 cm below the costal margin; and 7) an elevated hematocrit to >40% in female and >46% in male, which correspond to an increase of at least 20% in hematocrit from a gender-specific mean population baseline.

Severe dengue was defined by any of: 1) decompensated shock due to severe plasma leakage (defined as systolic blood pressure of <90 mmHg, a fall of ≥40 mmHg from a previous known baseline, or mean arterial blood pressure < 65 mmHg), 2) compensated shock due to severe plasma leakage (defined as clinical manifestations of reduced perfusion in presence of systolic blood pressure of ≥90 mmHg), 3) respiratory compromise due to severe plasma leakage, 4) severe hepatitis (defined as AST level > 1000 IU/L, or ALT level > 1000 IU/L), 5) severe bleeding that required intervention, or 6) severe organ involvement such as acute kidney injury defined by elevated serum creatinine, myocarditis or encephalopathy.

### Serology for dengue viral infection

All sera were tested for the presence of NS1 antigen and high-titre serum IgG. These were processed using the ELISA method (PanBio reagents). Our IgG is a high-titre IgG that is positive at titre >1:2560. IgM was not used in our study for 2 reasons. Firstly, it may remain positive weeks beyond an acute infection hence yielding a false-positive in a non-dengue infection, and secondly, to avoid false positive cases due to detection of cross-reacting antibodies.

### Measurement of biomarkers

As per standard practice at our centre, biomarkers were collected into appropriate Vacutainers®. CK samples were analyzed using the ultraviolet method, liver transaminases analyzed with International Federation of Clinical Chemistry method (without pyridoxal phosphate activation) and serum albumin were analyzed with bromocresol green (Roche Diagnostics, Malaysia). Platelets were analyzed with hydrodynamic focusing or direct current method, white blood count by flow cytometry and hemoglobin by photometrics (Sysmex, Malaysia).

### Composite indices

We formulated and tested several composite indices based on admission AST and admission ALT values in the search for a marker/model with good performance parameters that could help to better identify severe dengue. Composite indices, like body mass index and shock index [23], have been well-established in clinical use. We utilized simple and common mathematical operators e.g. squaring, division and multiplication in the formation of the indices. Simplicity was the core criterion in formulating these indices to allow for ease of clinical use. Fourteen indices were tested but only one, based on ease of calculation by the bedside, was further scrutinized. Though it is known that liver enzymes would vary along the course of illness, we made no attempt to adjust for this variation as it would only complicate deploying its use to the bedside.

### Statistical analysis

In a previous study [11], when investigators classified dengue into severe and non-severe dengue according to WHO 2009 classification, AUC of ROC curve obtained for AST was 0.62 (95% CI 0.57 - 0.67). Based on this information, we calculated that a sample size of 19 severe cases was needed for this study, estimating AUC of 72%, confidence level of 95% and power of 80%. No similar information was available for creatine kinase.

All data were analyzed using SPSS software (version 18.0; SPSS Inc., Chicago, IL). Continuous variables were tested for uniformity using the Kolmogorov-Smirnov test and normality with the Shapiro-Wilk test. Categorical variables were expressed as frequencies and percentages, and comparisons analyzed using the Chi-square test or Fisher’s exact test, as appropriate. As our data were mostly non-parametric, we used non-parametric analyses for data interrogation. Continuous variables with non-normal distribution were summarized as median and inter-quartile range (IQR) and comparisons were made using the Mann–Whitney U test. Correlation analyses to assess strengths of relationships between biomarkers were made by computing Spearman *r*. Predictive associations between biomarkers were analyzed using regression methods. The method yielding the highest *R* ^*2*^ was then reported.

Areas under curve of receiver-operating characteristic curves (AUROC) of all models (the composite indices, AST, ALT, CK and Hct) to identify at admission the occurrence of severe dengue, with their 95% confidence interval, were computed. As an alternative to validation using a second sample in assessing these models to identify severe dengue upon admission, we used the cross-validation technique. Cross-validation will avoid over-fitting of the models thus ensuring accuracy and validity.

The most accurate marker was then assessed further for its performance at four different cut-off values to achieve: 1) screening (cut-off 1), 2) optimal performance/Youden index (cut-off 2), 3) modified Youden to achieve higher specificity but maintain sensitivity >40% (cut-off 3), and 4) best diagnostic accuracy (cut-off 4). We assumed that these cut-offs would bear clinical relevance. Sensitivity, specificity, positive predictive value (PPV), negative predictive value (NPV), positive likelihood ratio (LR+), negative likelihood ratio (LR-) and diagnostic accuracy, with their 95% confidence intervals were calculated for that best marker. All tests of significance were 2-sided, and we took *p*-value <0.05 to indicate statistical significance.

## Results

### Patient characteristics

A total of 643 patients with suspected dengue who were admitted to the Department of Medicine, Kuala Lumpur Hospital between December 2014 and January 2015 were identified. Of this, patients were excluded for being NS1 antigen and high-titre IgG negative (169 patients) and younger than 18 years old (109 patients). The final number of eligible patients for final analysis was 365. The admission and in-ward characteristics of the patients are as shown in Tables 1 and 2. Among the patients, 28.9% were obese (BMI ≥27.5 kg/m^2^). Elevated admission levels of CK, AST, ALT and Hct occurred in 57.1%, 82.7%, 57.5% and 21.6% of patients, respectively. Elevated peak levels of CK, AST and ALT occurred in 80.6%, 92.2% and 81.9% of patients, respectively.

**Table 1:** Baseline characteristics and clinical parameter of 365 patients hospitalized with dengue

	All		Severe Dengue		Non-severe Dengue		*p*-value
	*n*	No. (% or IQR)	*n*	No. (% or IQR)	*n*	No. (% or IQR)	
Gender	365						0.003^*a*^
*Male*		215 (58.90%)	22	6 (2.80%)	343	209 (97.20%)	
*Female*		150 (41.10%)	22	16 (10.70%)	343	134 (89.30%)	
Age *(y), median (IQR)*	364	28 (23 - 36)	22	31 (23 - 36)	342	28 (23 - 36)	0.660^*b*^
BMI, *median (IQR)*	353	24 (21 - 28)	18	25 (21 - 29)	335	24 (21 - 28)	0.700^*b*^
Co-morbids present	365	55 (15.10%)	22	8 (36.40%)	343	47 (13.70%)	0.009^*a*^
*DM*	365	12 (3.30%)	22	3 (13.60%)	343	9 (2.60%)	0.003^*a*^
*Hypertension*	365	16 (4.40%)	22	3 (13.60%)	343	13 (3.80%)	0.006^*a*^
*Other co-morbids*	365	49 (13.40%)	22	6 (27.30%)	343	43 (12.50%)	0.100^*a*^
Number of co-morbids	365		22		343		0.003^*c*^
*0*		308 (84.40%)	22	14 (63.60%)	343	294 (85.70%)	
*1*		41 (11.20%)	22	5 (22.70%)	343	36 (10.50%)	
*2*		12 (3.30%)	22	2 (9.10%)	343	10 (2.90%)	
*3*		4 (1.10%)	22	1 (4.50%)	343	3 (0.90%)	
More than 1 co-morbids present	365	16 (4.40%)	22	3 (13.60%)	343	13 (3.80%)	0.006^*a*^

^*a*^ *Fisher’s exact test or Chi-square test, as appropriate*

^*b*^ *Mann-Whitney U test*

^*c*^ *Chi-square for trend*

*IQR* interquartile range, *BMI* body mass index, *DM* diabetes mellitus

**Table 2:** Laboratory parameters of 365 patients hospitalized with dengue

	All		Severe Dengue		Non-severe Dengue		*p*-value
	*n*	No. (% or IQR)	*n*	No. (% or IQR)	*n*	No. (% or IQR)	
Duration of febrile phase *(d)*	360	4.8 (3.8 - 5.8)	21	5 (4.1 - 7.3)	339	4.8 (3.8 - 5.8)	0.170^*b*^
Phase at admission, febrile	360	230 (63.90%)	21	16 (76.20%)	339	214 (63.10%)	0.250^*a*^
Day of presentation *(d)*	361	4.3 (3.2 - 5.3)	22	4.2 (3.1 - 5.1)	339	4.3 (3.2 - 5.4)	0.570^*b*^
NS1 Ag	356	288 (80.90%)	22	20 (90.90%)	334	268 (80.20%)	0.270^*a*^
High-titre IgG	360	160 (44.40%)	22	11 (50.00%)	338	149 (44.10%)	0.660^*a*^
Admission Hct, median (IQR), %	365	42 (39 - 46)	22	41 (37 - 43)	343	42 (39 - 46)	0.160^*b*^
Admission CK, median (IQR), IU/L	354	208 (103 - 409)	19	212 (116 - 300)	335	207 (103 - 424)	0.760^*b*^
Admission AST, median (IQR), IU/L	364	79 (47 - 135)	22	231 (97 - 564)	342	75 (47 - 128)	< 0.001^*b*^
Admission ALT, median (IQR), IU/L	365	51 (29 - 100)	22	122 (49 - 230)	343	49 (28 - 91)	0.003^*b*^
Nadir WBC, median (IQR), ×10^3^/μL	364	2.5 (1.9 - 3.6)	22	2.8 (2 - 3.8)	342	2.5 (1.9 - 3.6)	0.540^*b*^
Nadir Platelet, median (IQR), ×10^3^/μL	364	41 (21 - 67)	22	27 (10 - 49)	342	42 (22 - 67)	0.020^*b*^
Nadir Serum Albumin, median (IQR), g/L	326	32 (30 - 36)	21	27 (23 - 29)	305	33 (30 - 36)	< 0.001^*b*^
Peak CK, median (IQR), IU/L	103	429 (199 - 1044)	10	404 (171 - 766)	93	431 (206 - 1118)	0.430^*b*^
Peak AST, median (IQR), IU/L	338	123 (74 - 243)	21	585 (144 - 1098)	37	116 (73 - 223)	< 0.001^*b*^
Peak ALT, median (IQR), IU/L	340	86 (41 - 175)	22	225 (107 - 403)	318	81 (39 - 162)	< 0.001^*b*^

^*a*^ *Fisher’s exact test or Chi square test, as appropriate*

^*b*^ *Mann-Whitney U test*

^*c*^ *Chi-square for trend*

*IQR* interquartile range, *CK* creatine kinase, *AST* aspartate transaminase, *ALT* alanine transaminase, *WBC* white blood cell count, *AKI* acute kidney injury

### Severe vs non-severe dengue

In our cohort, 22 (6%) patients had severe dengue, including 1 (4.5%) patient with decompensated shock, 11 (50%) with compensated shock, 2 (9.1%) with respiratory compromise, 8 (36.4%) with severe hepatitis, 2 (9.1%) with acute kidney injury and 1 (4.5%) with encephalitis.

There were some differences in the characteristics of patients with severe and non-severe dengue (see Tables 1 and 2). Patients with severe dengue were significantly more likely to be female, 72.7% vs 39.1%, *p* = 0.003. These patients were also more likely to have co-morbidities, 36.4% vs. 13.7%, *p* = 0.009, and were more likely to have diabetes mellitus 13.6% vs. 2.6%, *p* = 0.03. Patients with severe dengue were more likely to have at least 1 co-morbidity (*p* = 0.003) but there was no significant difference in terms of presence of multiple co-morbidities (*p* = 0.06). Patients with severe dengue had significantly higher admission and peak levels of AST and ALT, with peak levels being higher than admission levels. Nadir levels of platelet and serum albumin were also lower in patients with severe dengue.

### Diagnostic value of CK, AST and ALT for identifying severe dengue

We evaluated the diagnostic value of CK, AST, ALT, Hct and the 14 formulated composite indices for identifying severe dengue at admission. Overall, the composite index AST^2^/ALT was the best performing marker in identifying severe dengue on admission. The AUROC for liver enzymes were moderate, with AST being better than ALT, whilst the AUROC for CK and Hct were poor (Table 3). The four cut-off values for AST^2^/ALT were: 98.1 (screening/highest sensitivity cut-off), 402.5 (optimal/Youden index), 653.2 (modified Youden index) and 1193 (highest diagnostic accuracy) (Table 4).

**Table 3:** Area under curve (AUC) of CK, AST, ALT, Hct, AST^2^/ALT and 13 other composite indices

Markers	AUC (95% CI)
CK	0.52 (0.40 - 0.64)
AST	0.78 (0.68 - 0.89)
ALT	0.69 (0.56 - 0.81)
Hct	0.59 (0.47 - 0.71)
AST^2^/ALT	0.83 (0.73 - 0.92)
AST/ALT	0.77 (0.67 - 0.87)
AST x ALT	0.74 (0.62 - 0.86)
AST^2^ x ALT	0.76 (0.64 - 0.87)
AST x √ALT	0.76 (0.64 - 0.87)
√AST x ALT	0.72 (0.60 - 0.84)
AST/√ALT	0.83 (0.73 - 0.92)
√AST/ALT	0.42 (0.30 - 0.55)
AST^2^/√ALT	0.81 (0.71 - 0.90)
AST^2^/ALT^3^	0.52 (0.41 - 0.63)
AST^3^/ALT	0.81 (0.72 - 0.91)
AST^3^/√ALT	0.80 (0.69 - 0.90)
AST^3^/ALT^2^	0.83 (0.73 - 0.93)
AST^4^/ALT^3^	0.82 (0.73 - 0.92)

*AUC* area under curve; *AST* aspartate transaminase; *ALT* alanine transaminase; *Hct* hematocrit; *CK* creatine kinase; *CI* confidence interval

**Table 4:** Diagnostic value of AST^2^/ALT to identify severe dengue based on AST & ALT upon admission

Cutoff	Sensitivity % (95% CI)	Specificity% (95% CI)	PPV (95% CI)	NPV (95% CI)	Diagnostic accuracy (95% CI)	LR+ (95% CI)	LR– (95% CI)
> 98.1	95.5 (77.2 - 99.9)	40.5 (35.3 - 45.9)	9.3 (6.2 - 13.9)	99.3 (96.1 - 99.9)	43.8 (38.8 - 49.0)	1.6 (1.6 - 1.6)	0.1 (0.02 - 0.8)
> 402.5	59.1 (36.4 - 79.3)	92.4 (89.1 - 95.0)	33.3 (20.6 - 49.0)	97.2 (94.8 - 98.5)	90.4 (87.0 - 93.0)	7.8 (6.5 - 9.3)	0.4 (0.4 - 0.6)
> 653.2	40.9 (20.7 - 63.7)	97.4 (95.1 - 98.8)	50.0 (29.0 - 71.0)	96.3 (93.7 - 97.8)	94.0 (91.0 - 96.0)	15.6 (9.2 - 26.6)	0.6 (0.5 - 0.7)
> 1193	31.8 (13.9 - 54.9)	99.4 (97.9 - 99.9)	77.8 (45.3 - 93.7)	95.8 (93.2 - 97.4)	95.3 (92.7 - 97.1)	54.6 (11.2 - 264.9)	0.7 (0.6 - 0.8)

*AST* aspartate transaminase, *ALT* alanine transaminase, *CI* confidence interval, *PPV* positive predictive value, *NPV* negative predictive value, *LR*+ positive likelihood ratio, *LR*– negative likelihood ratio

### Correlation and predictive associations between markers

Strength of relationships between markers as determined by Spearman *r* and their respective predictive associations as represented by *R* ^*2*^ are shown in Table 5. AST and ALT showed excellent correlation with each other, at admission and at peak values. In addition, admission AST showed a good predictive association with admission ALT (*R* ^*2*^ 0.79) but the association weakened at peak values (*R* ^*2*^ 0.66) despite better correlation. The correlation between liver enzymes and plasma leakage markers, hematocrit and serum albumin were weak to negligible, *r* < 0.10 and < −0.40. Liver enzymes also showed very poor predictive associations with nadir platelet and nadir serum albumin, *R* ^*2*^ 0.003 - 0.09.

**Table 5:** Correlation coefficients and coefficients of determination for CK, AST, ALT, markers of plasma leakage and platelet count

	n	Spearman *r* (95% CI)	*p*	*R* ^*2*^
Admission CK - Admission AST	364	0.37 (0.27 - 0.46)	<0.001	0.13^a^
Admission CK - Peak AST	206	0.28 (0.15 - 0.41)	<0.001	0.07^a^
Peak CK - Admission AST	104	0.29 (0.09 - 0.46)	0.003	0.06^a^
Peak CK - Peak AST	92	0.22 (0.03 - 0.39)	0.020	0.06^a^
Admission CK - Admission ALT	364	0.26 (0.16 - 0.35)	<0.001	0.02^b^
Admission CK - Peak ALT	216	0.11 (−0.02 - 0.24)	0.110	0.01^b^
Peak CK - Admission ALT	217	0.25 (0.06 - 0.42)	0.010	0.05^c^
Peak CK - Peak ALT	217	0.12 (−0.08 - 0.32)	0.240	0.02^c^
Admission CK - Admission Hct	364	0.24 (0.14 - 0.33)	<0.001	0.01^c^
Admission CK - Nadir Platelet	363	−0.22 (−0.32 - -0.12)	<0.001	0.01^c^
Admission CK - Nadir Serum Albumin	326	−0.04 (−0.15 - 0.07)	0.500	0.001^c^
Peak CK - Admission Hct	104	0.03 (−0.17 - 0.22)	0.800	0.004^c^
Peak CK - Nadir Platelet	104	−0.10 (−0.29 - 0.09)	0.300	0.01^c^
Peak CK - Nadir Serum Albumin	104	0.06 (−0.13 - 0.25)	0.540	0.01^c^
Admission AST - Admission ALT	365	0.86 (0.82 - 0.88)	<0.001	0.79^b^
Admission AST - Peak ALT	217	0.68 (0.60 - 0.75)	<0.001	0.54^b^
Peak AST - Admission ALT	206	0.66 (0.57 - 0.73)	<0.001	0.45^a^
Peak AST - Peak ALT	189	0.92 (0.89 - 0.94)	<0.001	0.66^b^
Admission Hct - Nadir Platelet	365	−0.14 (−0.24 - -0.03)	0.009	0.06^c^
Admission Hct - Nadir Serum Albumin	327	0.19 (0.08 - 0.29)	0.001	0.03^c^
Nadir Serum Albumin - Nadir Platelet	327	0.52 (0.43 - 0.60)	<0.001	0.14^b^
Admission AST - Admission Hct	365	0.05 (−0.06 - 0.15)	0.380	0.006^c^
Admission AST - Nadir Platelet	365	−0.34 (−0.43 - -0.25)	<0.001	0.04^b^
Admission AST - Nadir Serum Albumin	327	−0.32 (−0.41 - -0.21)	<0.001	0.09^c^
Peak AST - Admission Hct	206	−0.02 (−0.15 - 0.12)	0.800	0.01^c^
Peak AST - Nadir Platelet	206	−0.38 (−0.49 - -0.26)	<0.001	0.08^c^
Peak AST - Nadir Serum Albumin	200	−0.29 (−0.41 - -0.16)	<0.001	0.09^c^
Admission ALT - Admission Hct	365	0.09 (−0.06 - 0.15)	0.080	0.003^c^
Admission ALT - Nadir Platelet	365	−0.19 (−0.43 - -0.25)	<0.001	0.03^c^
Admission ALT - Nadir Serum Albumin	327	−0.16 (−0.41 - -0.21)	0.003	0.04^c^
Peak ALT - Admission Hct	217	−0.02 (−0.06 - 0.15)	0.810	0.01^c^
Peak ALT - Nadir Platelet	217	−0.17 (−0.43 - -0.25)	0.020	0.01^c^
Peak ALT - Nadir Serum Albumin	209	−0.13 (−0.41 - -0.21)	0.050	0.03^c^

^a^ *Power regression*

^b^ *Linear regression*

^c^ *Polynomial regression;*

*CK* creatine kinase, *AST* aspartate transaminase, *ALT* alanine transaminase, *Hct* hematocrit

CK showed a weak to negligible correlation with liver enzymes at both admission and peak values (*r* 0.11 - 0.37) (Table 5). The correlations between CK and plasma leakage markers, hematocrit and serum albumin were negligible (*r* < 0.30 and < −0.30). Similarly, the correlations between CK and disease course marker, platelet count, were also negligible (*r* < −0.30). CK showed very poor predictive associations with liver enzymes, leakage markers and disease course marker (*R* ^*2*^ 0.001 - 0.13).

Interestingly, we also found that there was a negligible correlation (*r* 0.19 (95% CI: 0.08 - 0.29), *p* = 0.001) and a non-existent predictive association (*R* ^*2*^ 0.03) between admission Hct and nadir serum albumin (Table 5).

## Discussions

Our analysis showed that admission CK was not helpful in identifying severe dengue and does not correlate with any of the biomarkers which represent hepatitis (ALT), plasma leakage (hematocrit and serum albumin) and disease course (platelet count). Musculoskeletal involvement in dengue takes the form of myalgia and arthralgia. It was demonstrated in a cohort of 1716 patients in Latin America that this occurred in 94.3% of their patients [24]. We emphasize again that our study focused on biomarkers at admission - when it would be most relevant time to predict development of severe dengue. Also, as mentioned, our case definitions used the current WHO 2009 guidelines. In Villar-Centeno et al. (2008), the values of CK in the dengue haemorrhagic fever (DHF, WHO 1997) group were mean (95% CI): 549.6 (267.5–831.6) U/L taken 2-4 days into the illness. Our study showed admission CK in the severe dengue group are median(IQR): 212 (116 - 300) U/L taken 3-5 days into the illness. The peak CK values in our study were 404 (171 - 766) U/L. The difference between non-severe and severe groups were not significant (*p* = 0.43). It seems therefore that although musculoskeletal involvement occurred in almost all dengue patients, its significance beyond symptomatology is unclear. Further studies are needed to demonstrate the clinical relevance of monitoring CK.

However, our study revealed that, at admission, the identification of severe dengue was best using the composite index, AST^2^/ALT, based on AST and ALT readings taken at admission. The AUROC was 0.83, which makes it to date one of the two markers with highest diagnostic performance. Our composite index requires, however, the performance of liver function tests and AST. At our hospital liver function tests and AST are available on an urgent basis with a short turnaround time. The simplicity of the formula allows for ease of clinical use. We propose 2 cut-offs, 402 and 653, that will help to delineate severe dengue. Such suspicion may arise in those who have, for example, isolated tachycardia in the presence of high grade fever, isolated single manifestation of peripheral sign of shock, a remarkable isolated derangement of hematocrit or isolated mild metabolic acidosis. Choice of cut-off will also depend on clinical resources available, with the higher value more suitable if resources are limited.

In addition, our correlational analysis suggests that AST originates from the liver rather than muscles. AST has been showed to rise earlier and to a higher level than ALT in almost all dengue patients [12]. This makes AST a better marker to monitor the liver and identify severe hepatitis earlier. Its levels also decline earlier than ALT [8], making it well-suited in monitoring the course of hepatitis. In comparison to other biomarkers and composite indices studied, the diagnostic performance of AST was second only to AST^2^/ALT, with AUROC of 0.78. Thus, it is better than the traditional ALT in monitoring hepatitis in dengue. Our results of the diagnostic performances of the liver enzymes are better than a previous study [11] because we included severe hepatitis as part of the outcome of severe dengue. We believe it is important to be able to identify the development of severe hepatitis at an earlier stage albeit without the patient manifesting other features of severe dengue.

Our correlational analysis found that the pathophysiological processes that occur in dengue, represented by the different biomarkers studied, do not correlate with each other, confirming our anecdotal observation. This suggests that these processes occur independently of one another in dengue. Adding to the puzzle is our findings that markers of plasma leakage, Hct and serum albumin, do not correlate with each other. The sampling time points of these biomarkers in our study were different. Nonetheless, two markers representing the same disease process ought to have a better degree of correlation. Furthermore, the sampling time points chosen in our analysis were appropriate for each hematocrit and serum albumin. Admission Hct would not be influenced by the later clinical administration of fluid and may be assumed to be the highest before intervention was instituted. Similarly, we used nadir serum albumin which may reflect peak leakage and is unaffected by fluid administration. This paradox needs further evaluation.

The main limitation of our study was that it was retrospective. However, data accuracy was reasonable as management of patients followed standard local management guidelines for dengue with clear specifications of timing of blood investigations. Our second limitation was that we did not perform serotyping. The dominant serotypes during the time of this study were Den-1 at approximately 55% of national surveillance cases and Den-2 at approximately 35%. Den-3 and Den-4 each contributed to less than 10% of cases. Since mid-2013 Den-1 and Den-2 have been the predominant serotypes in Malaysia [25]. Third, although we suggested that AST was of liver origin based on the strong correlation with ALT and negligible correlation with CK, we did not test for other sources of AST such as brain, red blood cells and kidney. However, none of our patient had hemolysis, only 2 patients had increments in serum creatinine and only one had encephalitis. Fourth, we did not perform multivariate analysis on our composite indices. A multivariate analysis would require a larger number of patients with severe dengue, which in turn would be determined by the number of covariates included. Finally, we did not adjust for the usage of paracetamol and traditional medication which is prevalent in our population, which could have been one of the causes of elevated transaminases. We have focused and limited our discussion to biomarkers examined in our study as per objective. There are of course many other known risk factors that are known to be associated with development of severe dengue, beyond the scope of our study. A larger study that examines extensively all potential risk factors together would be suited to review these.

## Conclusion

We conclude that AST^2^/ALT may be used as a marker to identify severe dengue based on admission AST and ALT, with two choices of cut-off values, 402 and 653.

## Abbreviations

ALT: Alanine aminotransferase
AST: Aspartate aminotransferase
AUROC: Area under the receiver operating characteristic
CI: Confidence interval
CK: Creatine kinase
ELISA: Enzyme-linked immunosorbent assays
Hct: Hematocrit
IgG: Immunoglobulin G
IgM: Immunoglobulin M
IQR: Inter-quartile range
LR–: Negative likelihood ratio
LR +: Positive likelihood ratio
NPV: Negative predictive value
NS1: Non-structural protein-1
PPV: Positive predictive value
ROC: Receiver operating characteristic
STARD: Standards for reporting of diagnostic accuracy
WBC: White blood cell count
WHO: World Health Organization.
